# The Global Historical Climatology Network Monthly Precipitation Dataset, Version 4

**DOI:** 10.1038/s41597-024-03457-z

**Published:** 2024-06-15

**Authors:** Scott Applequist, Imke Durre, Russell Vose

**Affiliations:** NOAA/National Centers for Environmental Information, Asheville, NC USA

**Keywords:** Hydrology, Hydrology

## Abstract

The Global Historical Climatology Network (GHCN) monthly precipitation dataset contains historical time series for thousands of land surface stations worldwide. Initially released in 1992 and revised in 1998, the dataset has been employed in a variety of applications over the past three decades, including operational monitoring, applied research, and international assessments. This paper describes the data and methods used to compile the latest edition (version 4), which has three major enhancements. The first enhancement is to the station network, which increased in size by a factor of five due to the inclusion of dozens of new source datasets, most notably GHCN Daily (GHCNd). The second improvement is the application of a rule-based algorithm to compare and merge records representing the same location. The third enhancement is to the quality assurance approach, now consisting of 18 new checks based on GHCNd and other operational systems. Updated monthly, the resulting dataset consists of time series of monthly precipitation totals at more than 120,000 worldwide stations, including more than 33,000 active observing sites.

## Background & Summary

The Global Historical Climatology Network (GHCN) is an integrated collection of global, regional, and national data sources from land surface stations worldwide. It consists of several datasets with different temporal resolutions and meteorological variables. For monthly precipitation totals, two major editions have been released thus far^1,2^, with the last minor process revision in 2005 and monthly updates since. Freely available from NOAA’s National Centers for Environmental Information (NCEI), this quality assured station dataset is used in climate change research and assessments (e.g., the IPCC Sixth Assessment Report^3^), establishing a ground truth for relationships between satellite observations and rain gauges^4^, and NCEI’s suite of Climate Monitoring services (e.g., https://www.ncei.noaa.gov/sotc/global/202213#precip), which place recent conditions into historical context. Furthermore, it is a key ingredient in the Global Precipitation Climatology Centre’s (GPCC) surface observation network, the underlying station database for the Global Precipitation Climatology Project (GPCP^5^), and thus supports the state-of-the-art analysis of worldwide precipitation.

The original GHCN^1^ consisted of a pair of datasets, one for monthly precipitation totals and one for monthly-mean temperature. With a goal of assembling long records at locations that would be suitable for climatological analysis over many decades, its creators sought to bring together disparate datasets in a unified format and apply basic quality control measures. In its second generation^2^, additional datasets were included to increase spatial and temporal coverage, and more rigorous quality control measures and a means to periodically update the precipitation and temperature datasets were applied. Since then, the monthly temperature and precipitation components of GHCN have evolved separately, and a complementary multi-element daily dataset (GHCNd^6^) was added to the GHCN family.

The purpose of this paper is to document the construction of the latest version of the GHCN monthly precipitation dataset. As with the first two versions of GHCN monthly precipitation, the process involves the themes of gathering robust data sources, eliminating duplication and joining records, and identifying those data points having dubious quality. The first part of the methods section provides an overview of the datasets that comprise the new version. While several of the sources used in previous versions have been retained, monthly totals based on GHCNd now serve as the primary data source.

Next, using techniques drawn from a similar data assembly problem in upper air data^7^, the process is presented by which station time series from the often overlapping data sources are compared and merged. These are based on similarities in their station identifiers (IDs), coordinates, and data. Matching and conflicting series in the component datasets are identified, and using these comparison results, series are retained, merged, or removed.

Finally, the enhanced quality assurance system is described. A sequence of tests inspired by those used on daily data^6^ are applied to identify data of suspect quality. Data points so identified are flagged and left in the data files for those users who may have corroborating evidence of their validity, or who wish to perform their own quality control.

The result is a dataset of monthly precipitation values, updated each month, with over 33,000 active stations, over 10,000 of which are outside the United States, available from NCEI at https://www.ncei.noaa.gov/products/land-based-station/global-historical-climatology-network-monthly. Data files are provided in a format that is both human- and machine-readable. The name of this dataset is Global Historical Climatology Network Monthly Precipitation, Version 4, henceforth GMP4. Version numbering has skipped 3 to align with the GHCN Monthly Temperature^8^ product.

## Methods

The meteorological data collected around the world comes from a variety of measurement and recording practices. As a consequence, there is no single repository for all such data, either for historical or on-going collections. At a given location there may be data from different collections for different periods of time, with or without overlap. Furthermore, essential metadata about these records, in particular their location name and coordinates, may differ in various respects, including the following:

- the location name and that name’s spelling, either in a native language or English translation
- the precision to which the latitude and longitude of the location are provided
- a presence or lack of observational network identifiers established by the country or an international body

All of these issues amplify the challenge of trying to assemble a dataset that provides a robust indication of how much precipitation fell across space and over time. To mitigate these issues on GMP4, accented characters were replaced with English letter equivalents in location names. Latitudes and longitudes reported as zero were set to a special missing value after confirming that the location’s name or country invalidates zero as a possible coordinate. Previously assigned identifiers such as World Meteorological Organization (WMO) or GHCN codes were used when available to establish a 11-character identifier using a 2-character country code, a single-character network code, and an 8-character station numbering specific to the network, as is convention in other parts of GHCN. For GMP4, a provisional GHCN ID was generated for each non-GHCN source record based on the appropriate country and WMO ID (if known), or an internal counter.

Across all source datasets the majority of distinct locations represented have either a single record or two records in good agreement with each other. In numerous cases, however, agreement among records that appear to apply to the same site is ambiguous. For these situations, a systematic approach for comparing multiple records and deciding which to merge and which to exclude from GMP4 is needed. What follows is an explanation of that process, along with a description of the quality assurance measures that were applied to create the final GMP4 product from the merged dataset.

### Data sources

The five data sources assembled into GMP4 are themselves collections of datasets. Each has its own format and means of providing station metadata, flagging trace and missing values, and providing other indications of data quality. Various customized preparatory steps were designed to stage these as a starting point in GMP4.

### GHCNd

The primary source for GMP4 is the GHCNd dataset, the world’s largest collection of daily data, which includes more than 118,000 quality-controlled station time series of daily precipitation measurements^6^. For GMP4, GHCNd values are summed to create monthly totals. Values already flagged as suspect in GHCNd are treated as missing. A month is allowed to have no more than five daily values missing. Multi-day accumulations are included when available and cover five or fewer days, but they are not used if they span a boundary between months. Meteorological service agencies of the United States, Canada, and Australia have contributed their most authoritative data to GHCNd. Consequently, with few minor exceptions, records for these countries from other sources are pre-emptively excluded.

During the calculation of monthly totals, a set of spatial consistency checks is performed on the number of precipitation days in the month^9^. A monthly total is flagged as erroneous if precipitation is recorded on many fewer or many more days for that station-month than at neighboring stations within 75 km. This check is particularly effective at identifying months in which missing precipitation values are incorrectly recorded as zero. When the number of days with precipitation in a month is grossly different from neighboring locations, that month’s value is marked as failing a spatial quality control test.

Full station history information is available through the Historical Observation Metadata Repository (https://www.ncei.noaa.gov/homr/) which reflects known changes over time. For the purposes of assembling this dataset, metadata at the most recent location is treated as constant throughout the period of record. GHCNd is reprocessed each day at NCEI with daily values for the last day of the preceding month typically available on the third day of the current month.

### U.S. Historical Climatology Network

The U.S. Historical Climatological Network (USHCN^10^) is a collection of 1,218 long term, high quality stations in the contiguous United States. Most of the data for these stations are already in GHCNd, but USHCN contains 165,114 monthly records that GHCNd does not. Some USHCN stations are composites of multiple, usually consecutive, GHCNd station records; to simplify the merging process later, these records were decomposed into the original component stations. Values that were estimated or interpolated in USHCN are treated as missing, as are values for which there is an indication that more than five days in the month were missing from the month’s precipitation total.

### International collection

The “international” collection is a static archive consisting of 43 constituent data sources previously included in GHCN version 2. The collection includes a variety of global, hemispheric, continental, and national-scale sources spanning the years 1697 to 2008. Data sources were acquired using a variety of strategies, such as contacting data centers, drawing upon personal contacts, conducting literature searches, and digitizing manuscript records^2^. The international collection contains 36,755 time series for potential inclusion in GMP4, many of which are duplicates reported from different sub-sources. (Because of the availability of other trusted sources, U.S., Canadian, and Australian stations from the international collection are withheld from GMP4).

### World Weather Records

The World Weather Records (WWR^11^) database contains historical monthly data from 5,424 land surface stations worldwide, 4,409 of which have precipitation data. First released in 1927, the WWR database has been widely employed in operational climate monitoring, international climate assessments, and numerous other applications. To date, there have been 11 editions of WWR, the first containing data up through 1920, with each successive release containing data for another decade starting with 1921–1930. Since its inception, WWR has been produced by three different institutions: the Smithsonian Institution (1927, 1934, 1947); the U.S. Weather Bureau (1959, 1967); and the U.S. National Oceanic and Atmospheric Administration (NOAA; 1983, 1991, 2005, and ongoing). WWR consists of monthly mean values of precipitation, temperature, and pressure supplied by National Meteorological Services of WMO members.

### Monthly Climatic Data for the World

Monthly Climatic Data for the World (MCDW^12^) is a multi-variable data collection produced by NCEI. Beginning in 1986, these monthly values are derived from CLIMAT^13^ messages, transmissions of monthly observations over the Global Telecommunications System from WMO member nations. The dataset is updated each month, though the records are often several months behind the present. In a 2019 revision, metadata was included with each month’s values.

### Data assembly

This section describes the methods used to assemble the dataset, achieving both goals of combining records into a longer historical time series when appropriate and eliminating duplicates. Here we define a “record” as a station time series from one of the five data sources described in the previous section.

One goal in assembling GMP4 was to create long records at locations that would be suitable for climatological analysis over many decades. This often meant combining shorter records for which the locations provided were not exactly the same, but which otherwise exhibit similarity (e.g., in their location names and/or data). Small location discrepancies among such records may be due to differences of precision in reporting the latitude and longitude coordinates or may reflect different nearby measurement sites. Consequently, the values in this dataset are meant to represent what occurred in the vicinity of the location given, especially in countries with less dense observation networks. In the final GMP4 dataset, the appropriate data source and location as reported by the source are included with each monthly precipitation value.

Making comparisons for the purpose of combining records also enables us to eliminate duplicate records for a location. If two records are meant to represent the same location, retention of both will bias any analysis that uses them.

### Comparison of records

As a first step, each source record is compared to all other time series. Metrics in four categories are computed to identify similar records: position, data, name, and ID. There are up to four possible outcomes, as defined below, for each element: Equivalent, similar, inconclusive, and different. The joint outcome of these comparisons feeds a decision process described in the next section which determines whether two source station records reflect data for the same or different locations.

#### Position

If both records have valid latitude and longitude coordinates, the distance between them along the surface of a sphere with radius 6,371 km is computed. Locations within 10 km are considered equivalent, thus allowing for roughly a tenth of a degree variation or rounding off of the coordinates. Locations that are separated by between 10 km and 100 km, are considered similar. Locations that are separated by more than 100 km are considered different. If either record is missing a coordinate, the distance result is inconclusive.

Since coordinates occasionally have sign errors, a second distance is also computed using the absolute values of the latitudes and longitudes. If this “second chance” distance is less than 100 km, the distance result is changed from different to inconclusive.

#### Data

For each pair of station time series, a data matching score is computed from the months they have in common. For each of these, at least one value must be more than a trace for a comparison to occur. If the values are identical, or if both values round to the same nearest whole millimeter, a full matching point is awarded. When both values are greater than 5 mm, a full point is awarded if the values are within 2% of each other, and a half point is given if they are within 10%. The percentage score is the number of points divided by the number of comparisons. When this score is greater than or equal to 80% and 60%, the matches are defined as equivalent and similar, respectively. When the percentage score is less than 40%, the data are declared to be different. Between 40% and 60% the comparison is inconclusive, as are cases when there are fewer than 6 overlapping months to compare or fewer than 6 months matching.

#### Location name

Location name strings are compared allowing for minor variations in spelling, standardization of abbreviations, non-alphanumeric characters, and spacing. The location names are considered equivalent if their strings (1) have the same number of space-delimited substrings, and (2) these substrings differ only by one character or contain the same consonant sequence. Otherwise the result is inconclusive. To avoid unintended matching, locations must have valid coordinates and a distance of less than 100 km between them. Illustrations of alternative spellings for Khorugh, Tajikistan and their results appear in Table 1.

**Table 1:** Examples of location name and ID comparisons and results.

First location name	First source	Second location name	Second source	Result
HOROG	Intl Coll	KHOROG	MCDW	equivalent
KHOROG	MCDW	KHORAG	WWR	equivalent
HOROG	Intl Coll	KHORAG	WWR	inconclusive
First location ID	First source	Second location ID	Second source	Result
TI000038954	GHCNd	TIMLP038954	WWR	inconclusive
TIMLP038954	WWR	XXMLP038954	MCDW	equivalent
TI000038954	GHCNd	XXMLP038954	MCDW	inconclusive

#### Location ID

If the 11-character station IDs are identical between records, they are said to be equivalent. The station IDs are also deemed equivalent when they contain the same WMO station identifier, as indicated by a network code (third character) of “M” and identical numbers in characters 7 to 11. As with location names, the comparison outcome can only be equivalent or inconclusive. Examples from Khorugh, WMO ID 38954, are shown in Table 1.

#### Additional rules for fine tuning the matching results

In general, the above-stated matching criteria led to obviously matching records being paired with each other and to clearly distinct records being kept apart. However, without further refinement, there were some instances in which conflicting comparison outcomes resulted either in source records for multiple locations being erroneously matched with each other or in the unfortunate removal of long, high-quality records. Consequently, the following additional measures are taken to reduce the number of conflicts among matching criteria and to improve the integrity of the final GMP4 station records.

- Preventing the merging of two GHCNd stations: As known independent records, two GHCNd sourced records are not allowed to merge if they overlap. To ensure this outcome later in the process, overlapping GHCN records are declared to have different data during the data comparisons.
- Encouraging merges of records at nearly identical locations: When two non-US records, at least one of which does not originate from GHCNd, are within 0.1 km of each other, and the station ID and location name comparisons are both inconclusive, the station ID comparison is set to a match in an effort to increase the likelihood that the records will be joined later.
- Removal of likely erroneous name matches: When two international collection records from the same sub source are within 100 km of each other and have an apparent location name match, this name match is set to inconclusive. Doing so resolves potential conflicts in the grouping stage when multiple station locations are given the same generic name of a city.

### Grouping

#### Verdicts for each pair of records

**Fig. 1:**
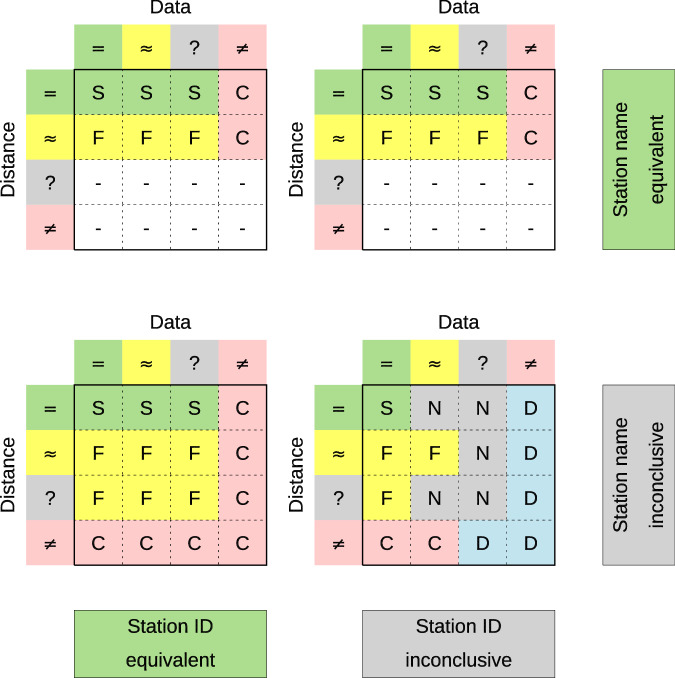
This collection of four tables shows how the four station matching tests come together to adjudicate station pairs. Symbols for distance and data matches indicate if the two records were equivalent (=), similar (≈), inconclusive (?), or different (≠). Since a station name match requires the stations to be within 100 km of each other, there can be no outcomes of inconclusive or mismatched distances in the upper tables. Descriptions of the five outcome sets of strong match (S, green), fair match (F, yellow), neutral (N, gray), conflicting (C, red), and distinct (D, blue) are given in Table 2.

The four categorical tests for record similarity described above yield a total of 48 different combinations of outcomes which are summarized in Fig. 1. These 48 outcomes can be organized into five sets according to their impact on the subsequent record grouping decisions. The definitions of these five sets of outcomes are listed in Table 2.

**Table 2:** Outcomes of combinations of the four categorical tests for record similarity.

Outcome	Key in Fig. 1	Description
Strong match	S, green	Records have multiple equivalent metrics indicating they go together.
Fair match	F, yellow	Records have multiple similar metrics indicating they go together. This designation is used as a tie-breaker below strongly matched pairs but typically has the same result.
Neutral	N, gray	Records lack any evidence that they should be either combined or kept separate. Their relationships to other records will determine how they are used.
Conflicting	C, red	Records have both equivalent (or similar) and different metrics that must be resolved.
Distinct	D, blue	Records have no metrics that are equivalent or similar. Records should be kept separate.

For the most part, the difference between Strong and Fair matches is inconsequential and leads to the same outcome; the difference only matters in certain tie-breaking situations. An obvious example of Conflict is when the station IDs indicate records are meant to be from the same location, but the coordinates provided, and hence the distance between the stations, indicate they are over 100 km apart. Similarly, conflict between records exists if the station names match but the data do not. When data and distance are both well matched, it is determined that the records match regardless of the outcomes of the station name and station ID comparisons.

The purpose of the Neutral verdict is illustrated by the case in which stations are close, their names and IDs do not match, and the data are similar. In this case it is not clear whether the records are from two nearby, yet different observing sites, or whether they originate from the same observations, but were processed differently before being used for GMP4. By returning a verdict of Neutral the algorithm uses the joint relationships among other records to decide if these records should be joined or remain separate.

A record with results of exclusively separate or neutral in all comparisons is kept separate from all other records. This is the case for about 2/3 of the input records. Of the over 50,000 records that remain, nearly 16,000 preliminary groupings are formed as some relationship is detected between their records.

#### Resolving the pairwise verdicts

Of these nearly 16,000 groups, about 9,500 contain only a pair of records. Of these, 91% are a strong match and 7.5% are a fair match. These are marked for merging in the next step. 1.5% of the pairs are in conflict, in which case we remove the shorter of the two. When more than two records form a group, they often all match to some degree and get marked for merging. This occurs in about 70% of the groups with 3 or more records. In the other 30%, there is a combination of records with strong or fair matches (indicating they should go together) and conflicting or distinct verdicts (indicating they should be kept apart). An algorithm eliminates records causing the most conflict while attempting to retain those most in agreement. This process was originally developed for merging upper air data records in the Integrated Global Radiosonde Archive (IGRA) version 2^7^. Among each group of records showing some kind of relationship, possible results are sub-groups that should be combined into a single station record, records that should be removed, and individual records that should remain separate. In the most extreme case, a preliminary grouping of 56 records in Costa Rica had 15 records removed and was ultimately divided into 10 sub-groups.

### Merging

Records marked for merging are assembled in a priority order based first on their source and then the year in which the record ends. The source priority is the same as the order in which the sources are presented in the Data Sources section, but with WWR and the international collection being equal. Within a source priority group, records with the more recent ending years are given preference over others. The location’s name, coordinates, and station ID for the purposes of general inventory come from the first source record within this prioritized order. Given the data from this first record, subsequent group members are used to fill in any missing values. The process is repeated throughout the merge groups.

During the course of processing, certain errors in the station metadata were discovered and addressed in an effort to avoid the removal of data due to the resulting conflicts. For example, in some cases, the comparison of two records for the same location inadvertently created a conclusion of “different” in the distance metric when one of the two records had an erroneous latitude. When the correct location could be independently verified using an atlas, erroneous values were set to missing in the appropriate metadata, so that in subsequent comparisons and processing, they yielded a result of “inconclusive,” thereby not creating a conflict.

To further limit the loss of significant data records as a result of erroneous data or metadata, cases in which multi-decadal records were removed from a mingle group were also examined. While in most instances the presence of other records meant that these removals had little impact, in 15 cases it was found that by preemptively eliminating select, usually less substantial, records from a group, the grouping process could be steered to retain more data and/or avoid merging data from distinct observing sites. For example, in Hong Kong, there were 12 records, all within 10 km of each other and several with identical coordinates. These were represented by WMO IDs 45004 and 45005 which represent two distinct sites. With no intervention, the process created a single record from 1947-present and eliminated a record dating back to 1853. Manual removal of two records uncoupled the link between the different WMO IDs, resulting in two records: One from 1963 to the present, and the other from 1853 to 2016.

### Quality control process

Following the strategy described by Durre *et al*.^14^, a sequence of tests was developed to detect those values for which a reasonable doubt exists of their validity or usefulness. Once a value is flagged, it is no longer included in subsequent tests, which further helps determine the order in which the tests are performed. For example, inclusion of a value in excess of the world record would skew any statistics generated for its record or comparisons with neighbors. Thus, these are flagged prior to searching for physical outliers. As a compromise must be struck between allowing suspect values to pass and misidentifying valid values as bad, the results of each test were manually examined and thresholds were adjusted until a false alarm rate of no greater than 20% was achieved.

### Sparse records

Three tests eliminate entire records providing very little overall data. First, records with two or fewer monthly values are excluded. Next, records with values limited to zero, trace, and a single positive value are eliminated. Finally, records containing values in less than 20% of the months in years with data are excluded. In all, this eliminates 5,627 of the merged station records available at this stage, 87% of which are from Community Collaborative Rain, Hail and Snow Network (CoCoRaHS^15^) sites, a part of GHCNd.

### Out of range values

The world record for monthly precipitation is 9,300 mm at Cherrapunji, India^16^. This provides an upper bound on all plausible values. Twenty-five values were found to exceed this. Two negative values were also found in the dataset and marked as out of range.

### Repetition of values

Within a record, sequences of 6 to 12 months which are repeated in another year are flagged. To prevent false positives at drier locations, these sequences are required to have a sum of at least 50 mm, and more than half of them must be non-zero.

While true streaks of a constant value may occur (e.g., when the same amount of precipitation happens to fall in two consecutive months), the likelihood that such a repetition of values is valid decreases with increasing streak length. Also, since the frequency of monthly precipitation totals is positively skewed, smaller totals are more likely to recur by chance than larger ones. Therefore, the threshold for what is considered to be an unacceptable streak length decreases as the value being repeated increases.

To take these factors into account, three types of streak checks are performed. In the month-to-month check, streaks are flagged when they exceed a length that depends on the value repeated. For example, streaks of six or more constant values 20 mm or greater are flagged. Shorter streaks of five, four, three, and two constant values have minimum amount thresholds of 50 mm, 50 mm, 100 mm, and 2,000 mm, respectively. As such, for constant values of 2,000 mm or more, no repetition in consecutive months is permitted. In the year-to-year streak check, the same thresholds are used to flag repetitions of values in the same calendar month across consecutive years. The third check flags streaks of zeroes that extend over 12 or more months at locations that have at least two years in their history with an annual precipitation total of 50 mm or more. In all of these checks, missing or previously flagged values do not interrupt a streak. Examples of these repeated values that were flagged are shown in Table 3.

**Table 3:** Illustrations of flagged repeated values in tenths of millimeters. Boldface values are flagged at the sites indicated for the following reasons: At Medeo, a sequence of 6 values is repeated from one year to the next. At Bangui Ville, the same values in excess of 100 mm are repeated in three different months over the course of 3–5 years. At Fazenda Bela Vista, first ten occurrences of the value 273.0 mm and then repeated zeros are flagged.

Year	Jan	Feb	Mar	Apr	May	Jun	Jul	Aug	Sep	Oct	Nov	Dec
KZXLP337235, Medeo, Afghanistan												
1932	270	360	800	1160	1500	540	450	300	420	630	590	390
1933	310	630	650	1220	1600	750	360	500	420	**630**	**590**	**390**
1934	**280**	**360**	**800**	1380	1950	2260	690	990	1120	**630**	**590**	**390**
1935	**280**	**360**	**800**	680	660	1890	1090	90	640	670	650	440
1936	130	450	410	1420	1970	620	860	730	460	430	420	80
CTM00064650, Bangui Ville, Central African Republic												
1913	220	100	400	2270	2080	1090	1180	600	890	2060	60	0
1914	700	70	1160	1340	1710	1340	1870	2260	**1920**	2040	1040	340
1915	220	430	1160	1320	1130	1530	1160	1390	**1920**	2290	1330	210
1916	160	900	380	1830	1330	1590	1250	1280	**1920**	**2040**	2550	310
1917	220	430	1000	1320	1380	1210	**1870**	2260	**1920**	**2040**	1040	510
1918	220	430	1160	810	1110	1340	**1870**	1000	**1920**	**2040**	1730	340
1919	220	180	240	1390	1710	850	**1870**	3680	1890	1630	1040	340
BR038905240, Fazenda Bela Vista, Brazil												
1986	0	0	840	0	0	0	0	0	0	0	0	764
1987	594	0	1929	624	**0**	**0**	**0**	**0**	**0**	**0**	**0**	**0**
1988	**0**	**0**	**2730**	**2730**	**2730**	**0**	**0**	**0**	**0**	**0**	2280	2280
1989	**0**	**0**	**2730**	**0**	**0**	**0**	**0**	**0**	**0**		**2730**	**2730**
1990	**0**	**0**	**0**	**0**	**0**	**0**	**0**	**0**	**0**	**0**	**0**	**2730**
1991	**2730**	**2730**	**2730**	**0**	**0**	**0**	**0**	**0**	**0**	**0**	**0**	**0**

A final unnatural repetition of values regularly appearing in the MCDW and international collection sources is a collection of a country’s values being identical from one month to the next. This is apparently caused when countries transmit the data one month at a time and accidentally send the same data in consecutive months. To check for this, within a country represented by at least 10 stations, when more than half of the stations have the same value from one month to the next, each of which is 1 mm or more, the identical values are flagged in both months.

### Isolated values

Any value that is separated by at least 18 months from all other values is flagged as an isolated value. Reports in such situations are often the result of data transmission or recording errors. The lack of continuity for these values raises questions about their accuracy.

### Temporal outlier checks

An outlier is any data point separated from other data points in a sample in such a way that one might question its validity. For monthly precipitation totals, several tests were devised to identify these unusual values. The first two of these examine the distribution of values within a single station’s record.

First, statistical pools of values spanning three consecutive calendar months in all available years are assembled. Those values in the center month that are greater than 5 mm and that are more than 10 times the pool’s 95th percentile value are flagged as statistical outliers. The pool must contain at least 30 values, over half of which must be greater than zero. This check is similar to that used in previous work^14,17^.

A second statistical outlier test checks for significant gaps in the distribution of values within a particular calendar month across all available years. The values are sorted from smallest to largest, and consecutive pairs of values in the sorted distribution are checked for an excessively large difference between them. If an unacceptably large gap is found, all values on the outer side of the gap are flagged as errors. Given the usually positive skewness of such distributions, this gap check is performed differently and with different sample size requirements in the lower and upper halves of the distribution.

In the lower half of any distribution containing at least 20 values, the ratio between two adjacent values is computed. If the ratio of the smaller to the larger is less than 0.05 and the larger value is 200 mm or greater, a gap has been detected. All values on the lower end of the gap are flagged. This test is especially useful in identifying dubious values of zero.

Similarly, in the upper half of a distribution with three or more values, the size of an unacceptably large gap between adjacent sorted values A and B is a function of the magnitude of A, the smaller of the two values. Specifically, a gap is detected between A and B if the following three empirically-determined conditions are met: 1) The difference between B and A is at least 1,000 mm. 2) The magnitude of B is more than twice the value of A. 3) The ratio of the gap, B-A, to A exceeds the value of the expression 4 – (B-A)/1000. The effect of this sliding scale is that a difference B-A constituting an unacceptable gap in a dry climate may be acceptable in a wetter climate. For example, if A = 3,000 mm, values for B in excess of 6,000 mm are flagged; these are more than twice as large as A. If A = 1,000 mm, B must be more than 3,000 mm, at least three times as large as A, to trigger a flag.

### Spatial outlier checks

A third outlier test compares a station’s value in a given month to those of its neighbors. Here values are identified that may be valid in the climatology of the station (e.g., a very dry month), but appear suspect when compared to the neighbors. Records with at least three neighbors within 100 km are compared to the neighboring values for spatial consistency. To avoid a directional bias, either the nearest of these neighbors must be within 10 km, or the maximum bearing angle between all neighbors must be greater than 120 degrees. There are three separate checks.

In the first, a zero, or very small value, is flagged as erroneous among much larger values when the value is 1 mm or less and the minimum of all neighbors is 50 mm or more. To allow for cases where two locations in close proximity have suspect small values among a larger number of neighbors, a second check, applied when there are 10 or more neighbors, flags pairs of small values when the group’s third lowest value (or 5th percentile, whichever is greater) is greater than 50 mm and more than 50 times larger than the values in question.

In a third check, values greater than 100 mm that are much larger than their neighbors are flagged when the ratio of the value to its largest neighboring value is greater than the maximum of 5 and (15 – 0.05*value). For example, a value of 101 mm would be flagged if its largest neighboring value were 10 mm or less. A value of 301 mm would be flagged if its largest neighbor were 60 mm.

### Quality-control results

Table 4 summarizes the number of quality control flags applied to individual values. Of 128,365 records, 103,197 did not have any values flagged, 19,541 have one or more flagged values, and 5627 were eliminated. The complete dataset contains nearly 40 million values with fewer than 0.5% being flagged.

**Table 4:** Number of occurrences in five categories of quality control flags.

Quality Control Test	Number of values flagged
Non-zero days discrepancy in GHCNd	26,703
Out of range	27
Duplicated values	98,824
Isolated values	4,346
Outliers	6,515
Total	136,416

## Data Records

### Accessing the data

The dataset upon which this article was based is archived in Figshare^18^. Monthly updates for the dataset, the station inventory, and specific technical information may be found via NCEI’s data access portal: https://www.ncei.noaa.gov/products/land-based-station/global-historical-climatology-network-monthly. From here users may download the entire dataset or browse the station inventory, product documentation, and individual station files.

Of particular significance is ghcn-m_v4_prcp_readme.txt which details the inventory and data file structures. The comma separated value data files are intended to be both machine- and human-readable.

Data processing begins on the fourth day of each month, by which time most of the U.S. daily data from the last day of the previous month has been received at NCEI. Results should be publicly available on the sixth day of the month. Additional data values continuously arrive at NCEI from around the world. These are incorporated in follow-up runs throughout the month as resources allow.

### Dataset characteristics

Of the nearly 40 million monthly values in the entire GMP4 collection, almost 85% are sourced from GHCNd. 13% come from the international collection, and the other sources contribute 1% or less each. Prior to 1900, 60% are from GHCNd, 32% from the international collection, and 8% from USHCN.

**Fig. 2:**
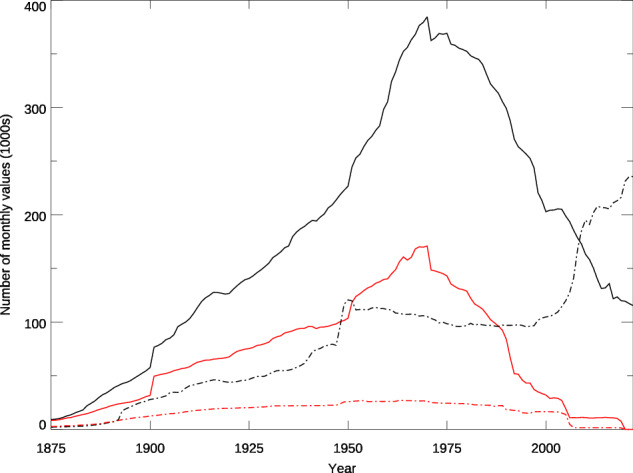
The number of monthly values in GMP4 (black) and GMP2 (red) for the world excluding the US (solid) and the US only (dashed).

Data in both GMP4 and GMP2 begin in 1697 with the same sets of observations from the Royal Botanic Gardens, Kew (United Kingdom). As shown in Fig. 2, the two versions have largely the same number of values until the late 1800s when GMP4 draws upon additional data sources and grows to more than twice the size of GMP2 by the time they both peak in 1970. Considering locations in the United States, GMP4 has 2 to 6 times as many values as GMP2 in the 20th century. The surge in U.S. values after 2000 is due to the inclusion of the rapidly expanding CoCoRaHS volunteer observer network that began in 1998. Declines in non-US data beginning in 1970 are punctuated at intervals coinciding with discontinued contributions from countries in the international collection.

For the entire period of record, GMP4 has 122,738 unique stations, approximately half of which are in the United States and its territories. GMP2 has 20,590 unique stations, 2,357 of which are located in the United States.

**Fig. 3:**
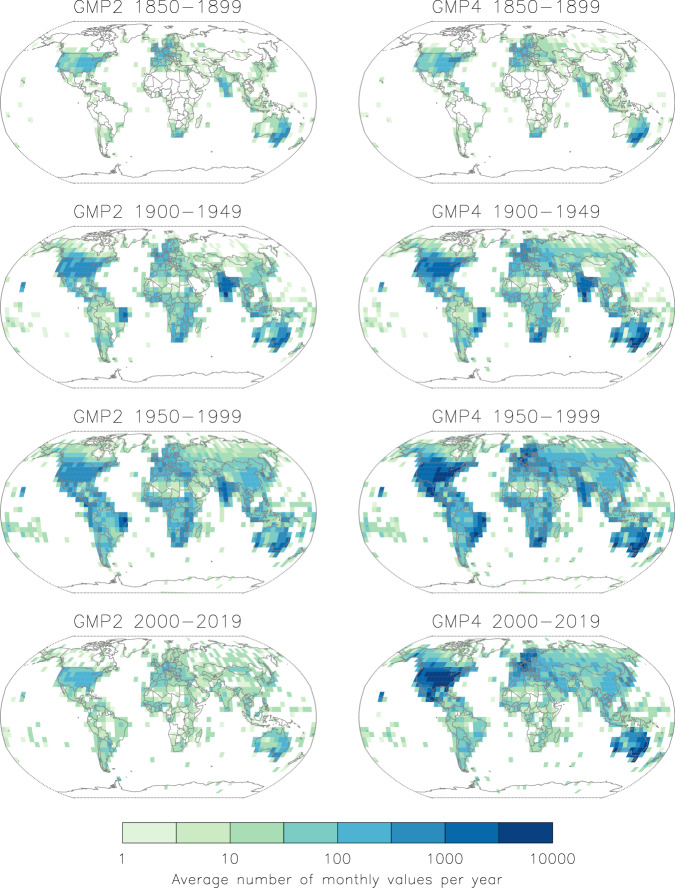
Depictions of the average number of monthly values per year within each 5-degree box for different blocks of years (1850–1899, 1900–1949, 1950–1999, 2000–2019) and for GMP2 vs GMP4. The scale is increasing dark shades of green, with every two increments corresponding to a power of 10.

Figure 3 shows the average number of monthly values per year during the year ranges given for each 5 × 5 degree box around the world. While the 1850–1899 period appears quite similar between GMP2 and GMP4, the subsequent periods show significantly more data availability in GMP4 for some of the more data sparse areas. While gaps in global coverage are still observed, GMP4 clearly adds more in all land areas except Greenland and Antarctica.

## Technical Validation

Demonstrating that the monthly values given in GMP4 are an accurate depiction of what happened is made difficult by a lack of independent precipitation datasets. However, even with shared sources, showing consistency with these illustrates the sound design and implementation of GMP4. In this section comparisons of GMP4 with its predecessor and with a high resolution global gridded dataset are presented.

### Comparisons with GMP2

Records from GMP2 and GMP4 were compared at 3,176 WMO stations around the world. These locations were selected because they are expected to have the best observing and data recording practices. Identifying pairs of records for comparison also involved computing the distance between their coordinates since countries may relocate WMO stations within their borders, and ensuring that there were six or more months in common between the two time series that had non-zero values. Having thus assured a distance and identifier match, these pairs of station records were compared using the data scoring method in the Comparison of records section above.

**Fig. 4:**
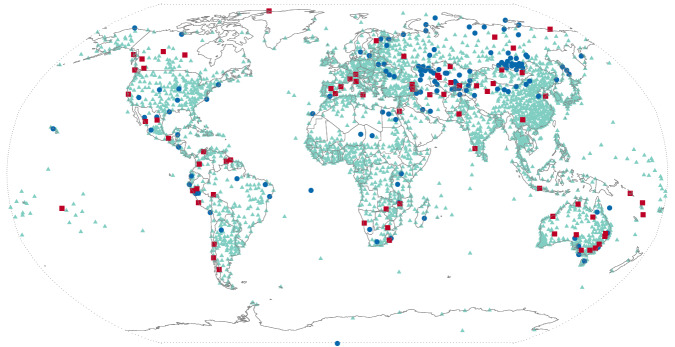
Locations compared between GMP2 and GMP4. Green triangles indicate a data match. Blue circles are inconclusive. Red squares indicate a data mismatch.

Perfect matches were found in 881 of the 3,176 pairs (28%). Another 40% matched almost perfectly, scoring between 90% and 99%. In all 93.5% of the pairs scored 60% or higher and would be deemed a data match. Inconclusive matches were found at 4.1% of the pairs, and 2.4% would have been declared a mismatch. These three data matching categories are plotted as different colored symbols at their locations in Fig. 4.

Examining the 10 worst of the mismatched cases can be as instructive as those that match well to illustrate that the GMP4 data integration process is working as intended. Reasons that the two datasets do not agree followed by the number of cases are:

- GMP2 used a data record that the GMP4 process removed due to discrepancies with other sources (three)
- The WMO identifier used in GMP2 was assigned to a neighboring GMP4 location with a distinct record (five)
- Errors in GMP2 assignment of WMO identifiers (two)

In summary, widespread agreement between GMP2 and GMP4 and explicable differences indicate that the newly developed, largely automated process for assembling the GMP4 dataset is working properly and is producing expected results.

### Comparison to MSWEP

As another level of comparison, point values from GMP4 are compared with those extracted from the Multi-Source Weighted-Ensemble Precipitation, version 2 (MSWEP^19^). MSWEP is a global, 0.1-degree resolution precipitation dataset that blends rain gauge, satellite, and reanalysis data. Selection of MSWEP was based on its strength when compared to radar data^20^ and its period of record allowing comparisons over the most recent three decades. Moreover, daily values from GHCNd are among the gauge data sources for MSWEP. Since nearly 85% of GMP4 is sourced from GHCNd, the fact that MSWEP and GMP4 share this data source was expected to help identify potential flaws in the GMP4 processing.

A total of 3,527 records from GMP4 that have at least 80% complete (non-missing and non-flagged) data from 1991–2020 were selected for comparison. To reduce over-representation of the more station-dense areas of the world, only the first location representing each whole latitude-longitude pair was evaluated. Values from the nearest 0.1-degree grid point to these stations were extracted from MSWEP monthly files.

**Fig. 5:**
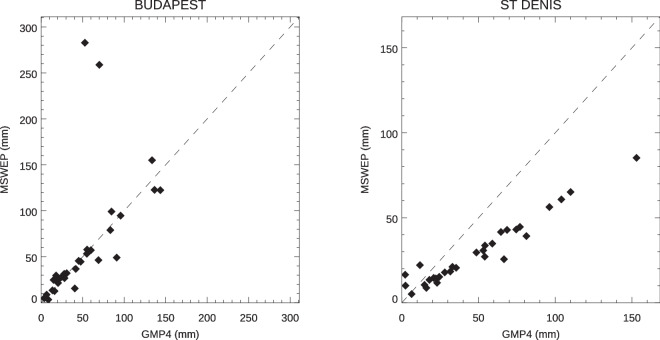
Scatterplots of September 1991–2020 precipitation values from GMP4 and MSWEP sources in Budapest, Hungary and St Denis, Reunion Island.

As some differences are expected, rather than using the strict data equivalence metric as before, the Spearman rank-order correlation, *r*_*s*_, was computed to measure how well the two time series match in a general sense. This metric reduces the impact of outlying values compared to the Pearson correlation coefficient, *r*^21^. Furthermore, unlike the normalized mean absolute error^22^, *nMAE* = ∑|*M*_*i*_ − *O*_*i*_|/∑*O*_*i*_, *r*_*s*_ does not penalize for systematic bias. Examples of these effects are shown in the panels of Fig. 5. In the first of these the values for Budapest, Hungary in September 1991–2020 from both GMP4 and MSWEP are plotted against each other. With the exception of two values, the values are near the *x* = *y* line. The values of *r* and *nMAE* for these are 0.58 and 0.42, respectively. However, *r*_*s*_ is 0.91. In the second plot, values for St. Denis, Reunion Island are plotted for the same period. There is nearly a factor of 2 difference between the precipitation values; *nMAE* is 0.44 while *r* and *r*_*s*_ are 0.97 and 0.93, respectively.

**Fig. 6:**
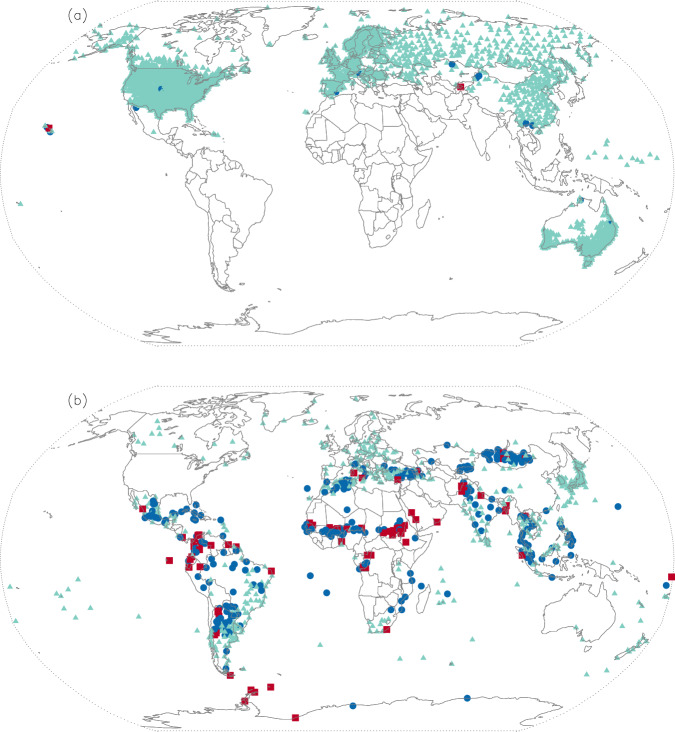
(**a**) 2608 Stations with 50% or more GHCNd as source. (**b**) 974 Stations with less than 50% GHCNd as source. Green triangles, blue circles, and red squares indicate a ranked correlation in the ranges greater than or equal to 0.75, between 0.5 and 0.75, and less than 0.5, respectively.

Across all stations evaluated, 72% of the *r*_*s*_ values were greater than 0.90 and 87% were greater than 0.80. Figures 6a and b depict *r*_*s*_ values for September 1991–2020, first at the locations for which GHCNd made up a majority of the 30-year records (Fig. 6a), and then at stations for which GHCNd was not the primary source (Fig. 6b). Since MSWEP and GMP4 both use, in large part, GHCNd values, it is no surprise that in Figure 6a 86% have *r*_*s*_ > 0.9. This accounts for 80% of the stations evaluated. In Figure 6b, the agreement is not as good, with 31% having *r*_*s*_ > 0.9 and 57% being greater than 0.8.

Investigations into these differences did not yield clear reasons for their presence. In Sudan the low scores appear at least in part due to a flooding event^23^ that lasted much of the rainy season in 2018. GMP4 contains values in Sudan for September 2018 that are in many cases 4–5 times their averages. It is unclear if these events, assuming the values GMP4 used from MCDW are accurate, were included as inputs to MSWEP, or if they were rejected by the MSWEP quality control process. Regardless, the cause of the differences westward across the Sahel, as well as in equatorial South America, Mongolia, and Antarctica is unknown. The greater weight given to reanalysis and satellite data in MSWEP for these regions is likely to contribute to the differences. It is clear, however, that GMP4 agrees with GMP2 (Fig. 4) in these areas of the world.

From these two analyses, one may conclude that where GMP4 is a reconstruction and extension of GMP2, it should be trusted equally if not more than GMP2. Furthermore, since GMP4 and MSWEP both source GHCNd and match well in those common areas of the world, the process of assembling GMP4 from the daily data is accurate as well.

## Usage Notes

As mentioned in the Data Records section, the most recent version of the dataset is available from NCEI’s website. The dataset archived in Figshare^18^ ends with values for November 2023.

As the records included in GMP4 expand with new data each month, and the entire period of record is reprocessed, a different path through the decision tree to merge or quarantine a small number of records may occur. This would result in changes to the final product.

Particular attention should be given to the quality control flag that may be present with each value. These values are included for completeness, but indicate a violation of one of the quality control rules. Users may wish to filter some or all of these flagged values, apply their own quality control methods, or entirely discard a station’s record with too many flagged values to be considered reliable.

Undetected errors in the data, such as shifts in measurement units, may remain. Attempts to develop an automated system for flagging suspected occurrences of such shifts were unsuccessful without also resulting in a large number of false positives.

Inhomogeneities undoubtedly exist within the dataset. These are easily noticeable within a record when the location or internal reference number provided with each monthly value changes. Others, such as those that may have been caused by changes to instrumentation or observation time within one of our original records, are not presently identifiable. As stated earlier, records are meant to be representative of a vicinity, and not a precise point. It is believed that these inhomogeneities should be within the noise level of the record provided.

## Data Availability

Code used for the dataset described here is available in Figshare^18^. Source code is archived with each month’s update per NCEI policy.
